# Autism Spectrum Disorder and Clinical High Risk for Psychosis: A Systematic Review and Meta-analysis

**DOI:** 10.1007/s10803-021-05046-0

**Published:** 2021-05-15

**Authors:** Julio Vaquerizo-Serrano, Gonzalo Salazar de Pablo, Jatinder Singh, Paramala Santosh

**Affiliations:** 1 Department of Child and Adolescent Psychiatry, Institute of Psychiatry, Psychology and Neuroscience, King’s College London, 16 De Crespigny Park, London, SE5 8AF UK; 2 Centre for Interventional Paediatric Psychopharmacology and Rare Diseases (CIPPRD), National and Specialist Child and Adolescent Mental Health Services, Maudsley Hospital, London, UK; 3 Early Psychosis: Interventions and Clinical-Detection (EPIC) Lab, Department of Psychosis Studies, Institute of Psychiatry, Psychology and Neuroscience, King’s College London, London, UK; 4 Institute of Psychiatry and Mental Health, Department of Psychiatry, Hospital General Universitario Gregorio Marañón Instituto de Investigación Sanitaria Gregorio Maranón, Universidad Complutense, Centro de Investigación Biomédica en Red Salud Mental (CIBERSAM), Madrid, Spain

**Keywords:** Autism spectrum disorders, Clinical high-risk for psychosis, Psychosis, Prodromal psychosis, Systematic review, Meta-analysis

## Abstract

Psychotic experiences can occur in autism spectrum disorders (ASD). Some of the ASD individuals with these experiences may fulfil Clinical High-Risk for Psychosis (CHR-P) criteria. A systematic literature search was performed to review the information on ASD and CHR-P. A meta-analysis of the proportion of CHR-P in ASD was conducted. The systematic review included 13 studies. The mean age of ASD individuals across the included studies was 11.09 years. The Attenuated Psychosis Syndrome subgroup was the most frequently reported. Four studies were meta-analysed, showing that 11.6% of CHR-P individuals have an ASD diagnosis. Symptoms of prodromal psychosis may be present in individuals with ASD. The transition from CHR-P to psychosis is not affected by ASD.

**Supplementary Information:**

The online version contains supplementary material available at 10.1007/s10803-021-05046-0.

Autism spectrum disorders (ASD) are early-onset neurodevelopmental disorders categorised by persistent deficits in social communication and restricted and repetitive patterns of behaviour (American Psychiatric Association, 2013). ASD and psychotic disorders have symptomatic overlap and were historically considered related conditions (Kanner, 1965; Ornitz & Ritvo, 1968). Initially, ASD was viewed as an early manifestation of psychosis (Bleuler, 1911; Kanner, 1943); nevertheless, autism and psychosis have been categorised as separate conditions (Kolvin, 1971) from the third edition of the Diagnostic and Statistical Manual of Mental Disorders, (DSM-III) (American Psychiatric Association, 1987), onwards.

ASD and psychotic disorders represent disabling neurodevelopmental conditions with marked deficiencies in social functioning, which may coexist more frequently than would be expected by chance (Davidson et al., 2014; Selten et al., 2015). Both conditions are highly heterogeneous, involving many complex features (Crespi & Badcock, 2008), and have been found to share several risk factors (Chisholm et al., 2015).

Increased paternal age (Hamlyn et al., 2013); obstetric complications (Hamlyn et al., 2013); specific genetic pathways (Burbach & van der Zwaag, 2009; Kushima et al., 2018); abnormalities in brain development (Toal et al., 2009); neuroanatomical markers (Toal et al., 2009), and social cognition deficits (Sugranyes et al., 2011a) are risk factors for ASD and psychosis. Additionally, a recent meta-analysis found that a diagnosis of ASD is a risk factor for developing psychosis (Lai et al., 2019; Zheng et al., 2018), with individuals with ASD being 3.5 times more likely to develop psychosis than the general population (Zheng et al., 2018).

Disorders on the autism spectrum and psychotic disorders might be considered as two extremes of the ASD-Psychosis continuum based on social cognition (Crespi & Badcock, 2008), and regulated by alterations in genomic imprinting (Badcock & Crespi, 2006), anatomical structure, as well as the function of the interacting amygdala, hippocampus, and prefrontal cortical circuitry (Baron-Cohen & Belmonte, 2005; Burns, 2004, 2006; Gisabella et al., 2005). With regards to neuroanatomical markers, and considering brain size, the existing evidence suggests that the amygdala and hippocampus is larger in ASD during early development compared to controls (Schumann et al., 2004), although this pattern is largely gone by adolescence and adulthood (Cheung et al., 2010; Courchesne et al., 2007). Further, there is evidence that indicates overgrowth of the brain in very young children at high risk of psychosis (Gilmore et al., 2010). Conversely, the brain is smaller in psychotic disorders during adulthood (Aleman & Kahn, 2005; Geuze et al., 2005; Gur et al., 2007; Kuroki et al., 2006).

ASD and psychotic disorders are considered to be influenced by dysregulated development of the social brain (Abu-Akel et al., 2015; Broks, 1997; Burns, 2006; Emery, 2000; Pourcain et al., 2018; Ziermans et al., 2020). It has been suggested that ASD and psychosis represent extremes on a continuum of human cognitive architecture from mentalistic cognition, (for example, theory of mind), to mechanistic cognition, (i.e. interaction with the physical environment) (Badcock, 2004; Crespi & Badcock, 2008). This model suggests that ASD and psychotic disorders represent opposite extremes of a social cognition continuum (Abu-Akel, & Bailey, 2000; Crespi & Badcock, 2008), in which ASD is associated with under-active mechanistic social cognition, and psychotic disorders with hyper-active mentalistic social cognition, diverging in opposite directions from typical performance (Abu-Akel, & Bailey, 2000; Abu-Akel et al., 2015). In terms of social cognition, it is known that social cognition is altered in ASD, especially in social personal interactions (Baron-Cohen & Belmonte, 2005; Bishop-Fitzpatrick et al., 2017; Rosello et al., 2020), whereas in psychotic disorders, especially in schizophrenia spectrum disorders, the abnormality is different and focussed on paranoid interpretation (Harrington et al., 2005b). In this line, a specific link between paranoid delusions and a deterioration of the theory of the mind has been suggested (Harrington et al., 2005a).

There has been an increasing interest in the overlap of ASD and prodromal symptoms of psychosis (Chisholm et al., 2015; Sampson et al., 2020). The shared clinical features among both conditions include unusual thought content, deficits in social interaction, and stereotyped behaviours (De Crescenzo et al., 2019; Hommer & Swedo, 2015). Furthermore, various studies have reported social cognitive deficits, a core symptom in ASD, in individuals at risk for psychosis (Lavoie et al., 2013; Lee et al., 2015). Several models have been suggested in computational neuroscience that helps to frame the basic brain-based mechanisms that might lead to the phenomenology of social cognitive dysfunction and the dimensions of the most common symptoms in individuals diagnosed with psychosis (Adams et al., 2013; Iglesias et al., 2017; Vladusich, 2008). Cognitive mechanisms proposed for psychotic symptoms, such as hallucinations, include a suggested abnormal perception resulting from an imbalance between (i) higher-order information processing, (i.e. perceptual expectations or previous knowledge), and (ii) lower-order perceptual processing of external sensory information (Aleman et al., 2003), and those experiences might be formed when higher-order cognition is prioritised over the lower-order sensory information (Hoffman et al., 2007; Hugdahl, 2009). Similarly, some symptoms present in ASD might result from an imbalance between top-down and bottom-up perceptual processing (Palmer et al., 2015; Van de Cruys et al., 2014). In this line, some psychotic symptoms might be explained in terms of a failure of top-down predictions (Adams et al., 2013). or an enhanced weighting of bottom-up prediction errors (Horga et al., 2014). Conversely, individuals with ASD are exceedingly influenced by a lower-order sensory information processing (Grossberg & Seidman, 2006).

The Clinical High-Risk state for psychosis designation (hereinafter CHR-P), also known as the “At-Risk Mental State”, (ARMS) (Schultze-Lutter et al., 2011), is defined as a status that confers high, but not inevitable risk, of developing psychosis, which implies that psychotic-like symptoms do not invariably lead to an acute psychotic episode (McGorry & Singh, 1995; Yung et al., 1996), describing individuals presenting with potentially prodromal symptoms (Fusar-Poli et al., 2013a, b). In terms of conversion to psychosis, the proportion of individuals at CHR-P who develop a psychotic disorder has been reported to be 22% at 3 years in a recent meta-analysis (Fusar-Poli et al., 2020a).

The CHR-P includes the (i) Attenuated Psychosis Syndrome (APS), defined as the presence of sub-threshold positive psychotic symptoms for at least 1 month during the past year; (ii) Brief Limited Intermittent Psychotic Symptoms (BLIPS), which is an episode of frank psychotic symptoms that spontaneously end within a week, and (iii) Genetic Risk and Deterioration Syndrome (GRD), which requires a significant deterioration in functioning for at least 1 month within the last 12 months, and a family history of a first-degree relative with a psychotic disorder or schizotypal personality disorder (Fusar-Poli et al., 2014a, 2016, 2020a, b; McGlashan et al., 2019; Salazar De Pablo et al., 2020) (eSupplementary Table 1).

Additionally, Basic Symptoms, considered as an immediate symptomatic expression of the neurobiological processes underlying psychosis, have been reported in individuals at CHR-P (Schultze-Lutter & Theodoridou, 2017; Schultze-Lutter et al., 2012). Basic Symptoms are defined as subtle subclinical disturbances in mental processes, such as affect, thinking, speech, perception, motor action and central-vegetative functions, with full insight into their abnormal nature (Schultze-Lutter & Theodoridou, 2017; Schultze-Lutter et al., 2012) (eSupplementary Table 2).

To our knowledge, this is the first systematic review, complemented by meta-analytical evidence, that comprehensively assesses the association between ASD and CHR-P. The aims of this study were (i) to describe the relationship between ASD and CHR-P; (ii) to understand the distinctive and overlapping features of ASD and CHR-P, including clinical, cognitive and pharmacological aspects; (iii) to provide evidence of the presence of ASD in CHR-P, and (iv) to conduct a meta-analysis into the co-occurrence of ASD and CHR-P.

## Methods

This study was conducted in accordance with the Preferred Reporting Items for Systematic Reviews and Meta-analyses (PRISMA) reporting guideline (Page et al., 2021), and the Meta-analysis of Observational Studies in Epidemiology (MOOSE) reporting guideline (Stroup et al., 2000) (eSupplementary Table 3). The study protocol was registered in PROSPERO (CRD42020183153).

### Search Strategy and Selection Criteria

A multistep literature search was performed by two independent researchers (JVS and GSP) through Pubmed and Web of Science database (Clarivate Analytics), incorporating the Web of Science Core Collection, BIOSIS Citation Index, KCI-Korean Journal Database, MEDLINE, Russian Science Citation Index, SciELO Citation Index, Cochrane Central Register of Reviews and Ovid/PsychINFO databases from inception until 5th October 2020, using the following keywords: (“Risk” OR “Prodrom*” OR “Ultra-High Risk” OR “Clinical High Risk” OR “Attenuat*” OR “APS” OR “High Risk” OR “Brief Limited” OR “Brief Intermittent” OR “BLIPS” OR “Genetic High Risk” OR “GRD” OR “At Risk Mental State”" OR “Risk of Progression” OR “Progression to First-Episode” OR “Basic Symptoms”) AND (“Psychosis” OR “Schizophrenia” OR “Schizoaffective” OR “First Episode Psychosis” OR “FEP”) AND (“Autism” OR “Autis*” OR “Autism Spectrum Disorders” OR “Autistic Disorder” OR “ASD” OR “Asperger Syndrome” OR “Asperger” OR “Pervasive” OR “PDD” OR “Childhood Disintegrative Disorder” OR “CDD”).

We also searched the preprint servers medRxiv and PsyArXiv from inception until 5th October 2020, using the keywords *“Autism” AND “Clinical High Risk” AND Psychosis”*. Additionally, the references of prior studies that were retrieved, were manually searched. Abstracts of articles identified that were not relevant were screened out. The remaining full-text articles were then assessed for inclusion eligibility against the inclusion and exclusion criteria.

### Eligibility Criteria

#### Inclusion Criteria

Studies included were: (1) individual studies, including abstracts, conference proceedings or grey literature; (2) in (i) CHR-P individuals (i.e., individuals meeting clinical-high-risk, prodromal psychosis and/or basic symptoms criteria as established by validated psychometric instruments), in whom the presence of ASD is reported, (ii) individuals with ASD in whom the presence of CHR-P is reported, and (iii) in which the overlapping and distinctive features between ASD and CHR-P are described, providing relevant data on the relationship between both conditions; and (3) published in English (eSupplementary Methods 1–2).

For the meta-analysis, additional inclusion criteria were: (1) reporting meta-analysable data, and (2) non-overlapping samples. Overlap was actively searched in the included studies by looking at the country, setting, university and program from which the sample was obtained, as well as the recruitment period. When more than one study from the same sample was detected, the study with the largest sample was included.

#### Exclusion Criteria

The exclusion criteria used were: (1) reviews, clinical cases and study protocols; (2) studies that did not formally assess and select participants at CHR-P or with ASD, and (3) studies written in languages other than English.

### Outcome Measures and Data Extraction

Data were independently extracted by two researchers (JVS and GSP), and discrepancies were resolved through consulting a third senior academic (PS). The variables extracted included: study (first author and year of publication); study design, (cross-sectional, longitudinal, clinical trials); setting (program or department); country; sample size; age (mean, SD); sex (% males); assessment instruments; comorbidity (if applicable); treatment received, (if applicable); key findings, and quality assessment.

### Quality Assessment

Study quality was assessed in all in the included studies. Though quality assessments can be reliably conducted in meta-analyses of experimental research, their use in observational studies is controversial, with no clear consensus on rating methods or their appropriate use in the analysis (Jüni et al., 1999). A modified version of the Newcastle–Ottawa Scale which has been used in recent meta-analyses in the field (Fusar-Poli et al., 2015; Salazar De Pablo et al., 2020) was used for the evaluation of cross-sectional and longitudinal studies, (www.ohri.ca/programs/clinical_epidemiology/oxford.asp). Scores ranged from 0 to 8 (eSupplementary Table 4).

### Data Synthesis and Meta-analysis

We systematically reviewed the available evidence on the relationship between ASD and CHR-P, focusing on distinctive and overlapping features, including clinical, psychopathological, therapeutic, cognitive and neurobiological aspects. The primary outcome was the presence of ASD in CHR-P individuals, (%, SE). Because the studies in this metanalysis were expected to be heterogeneous, the random-effects model was used. (DerSimonian & Laird, 1986 Heterogeneity among studies point estimates was assessed with the Q statistic. The magnitude of heterogeneity was evaluated with the I-squared index (Lipsey & Wilson, 2001). Publication bias was examined by visually inspecting funnel plots (Sterne et al., 2001) and applying the regression intercept of Egger (Egger et al., 1997). Due to the limited number of studies available, we could not test moderating factors using meta-regression analysis to evaluate sources of heterogeneity, in line with previous meta-analyses, that perform these analyses when at last ten studies per outcome are available (Salazar De Pablo et al., 2020). All p-values reported in the meta-analysis were two-sided and the level of significance was set at a p-value of less than 0.05. We used Comprehensive Meta-analysis Software, version 3 (Biostat, Inc) (Borenstein et al., 2005).

## Results

### Database

**Fig. 1:**
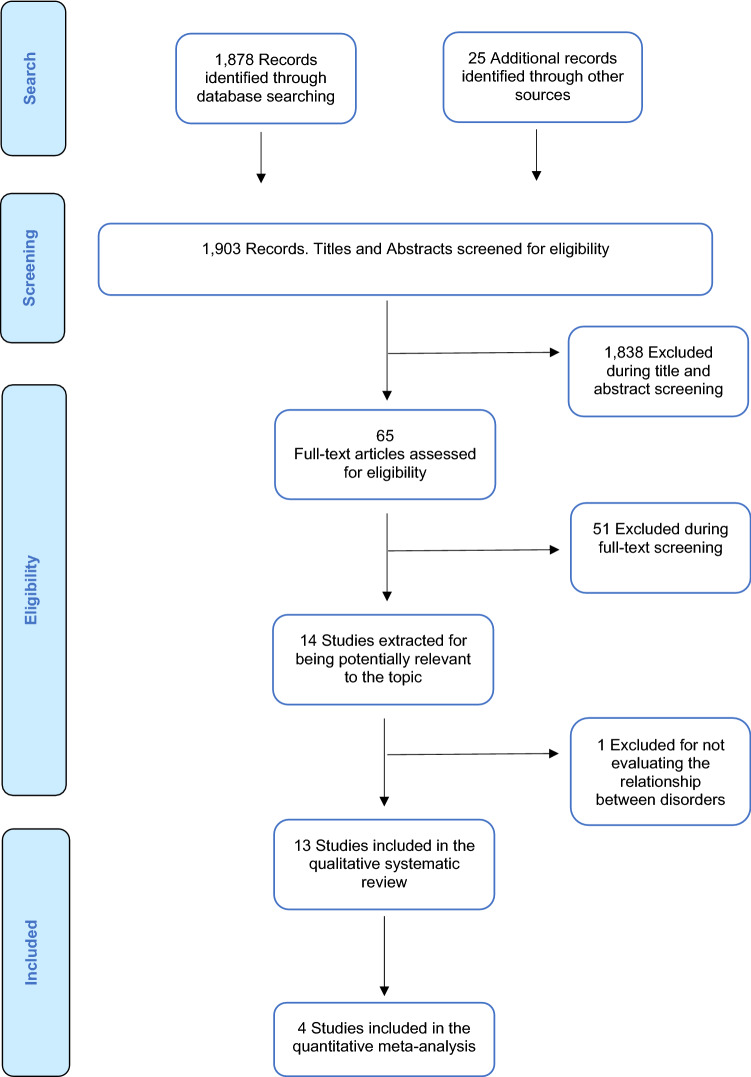
PRISMA flow diagram

The literature search yielded 1903 citations which were screened for eligibility. Of those, 1838 were excluded during the title and abstract screening, and 65 articles were assessed full text. This process resulted in a total of 13 studies being integrated into the current systematic review, which included a total of 16,474 individuals after removing duplicates. The data available allowed us to conduct quantitative meta-analyses of the proportion of CHR-P individuals fulfilling ASD criteria. After excluding overlapping samples and those studies that did not provide meta-analysable data, four studies were included in the meta-analysis on the prevalence of ASD in CHR-P (Foss-Feig et al., 2019; Maat et al., 2020; Solomon et al., 2011; Sprong et al., 2008) (Fig. 1 PRISMA Flowchart).

### Study Characteristics

The characteristics of the included studies are presented in Table 1. Nine studies (69.2%) were from the US (Foss-Feig et al., 2018, 2019; Guillory et al., 2018a, b; Jutla et al., 2020a, b; Solomon et al., 2008, 2011; Wilson et al., 2020), three (23.1%) from Europe (Eussen et al., 2014; Maat et al., 2020; Sprong et al., 2008) and one (7.7%) from Australasia (Fraser et al., 2008). All 13 studies reported on general characteristics of ASD and CHR-P state. Considering the measures to assess the CHR-P status, eight studies used the Structured Interview for Psychosis-Risk Syndromes (SIPS) (Foss-Feig et al., 2018, 2019; Guillory et al., 2018a, b; Maat et al., 2020; Solomon et al., 2011; Sprong et al., 2008; Wilson et al., 2020); four used the Scale of Prodromal Symptoms (SOPS) (Foss-Feig et al., 2018, 2019; Guillory et al., 2018a, b); one study the Comprehensive Assessment of At Risk Mental States (CAARMS) (Eussen et al., 2014); one used the Prodromal Questionnaire (PQ) (Eussen et al., 2014); two the Prodromal Questionnaire-Brief Child version (PQ-BC) (Jutla et al., 2020a, b); two the Kiddie-Formal Thought Disorder Rating Scale (KFTDS) (Eussen et al., 2014; Solomon et al., 2008), and one study the Bonn Scale for the Assessment of Basic Symptoms-Prediction List (BSABS-P) (Sprong et al., 2008).

**Table 1:** Main characteristics of the included studies addressing ASD and CHR-P

Study	Study type and design	Country	Sample size	Diagnostic criteria	Mean age (SD)	Sex % males	NOS	Key findings
Eussen et al. (2014)	Longitudinal	The Netherlands	142 ASD 32 CHR-P	ASD: DSM-IV/ADOS CHR-P: KFTDS/PQ/CAARMS	16.03 (1.97)	90,1	7	Illogical thinking predicted severity of ASD symptoms but did not predict prodromal symptoms of psychosis. Illogical thinking significantly predicted a higher total score on the ADOS symptom severity score. Loose associations did not correlate significantly with ADOS total scores. Perceptual disturbances were present in 43%, unusual thought content in 40% and non-bizarre ideas in 37.5%
Foss-Feig et al. (2018)	Longitudinal	US	305 CHR-P 14 ASD	ASD: DSM-IV CHR-P: SIPS/SOPS	n/a	n/a	3	CHR-P/ASD+ and CHR-P/ASD− did not differ in the rate at which they converted to psychosis. Individuals with ASD and CHR-P had greater social deficits but showed similar psychotic symptoms and have similar risk for conversion to psychosis
Foss-Feig et al. (2019)	Longitudinal	US	764 CHR-P 26 ASD 279 HC	ASD: DSM-IV CHR-P: SIPS/SOPS	18.53 (4.20)	57,06	6	Individuals with CHR with and without a comorbid ASD diagnosis did not differ in any of the summary scores of the SOPS domains. The CHR-P/ASD+ sample presented with higher levels of social anhedonia. CHR-P/ASD+ group had significantly lower social functioning scores compared to the CHR-P/ASD− group. Patients with CHR-P and ASD tended to exhibit greater social cognitive difficulties. Risk for conversion to co-occurring psychosis was equivalent between both groups
Fraser et al. (2008)	Cross-Sectional	Australia	n/a	ASD: DSM-IV	n/a	n/a	3	The prevalence rates of ASD in CHR and FEP cohorts were greater than current community-based estimates. There was a greater percent of FEP with ASD (4.25%) as compared to the CHR-P cohort (1.12%)
Guillory et al. (2018b)	Longitudinal	US	305 CHR-P 14 ASD	ASD: DSM-IV CHR-P: SIPS/SOPS	n/a	n/a	3	P300 amplitude to visual distractor stimuli was larger in CHR-P/ASD+ converters than those at CHR-P without ASD
Guillory et al. (2018a)	Longitudinal	US	305 CHR-P 14 ASD	ASD: DSM-IV CHR-P: SIPS/SOPS	n/a	n/a	3	CHR-P/ASD+ converters showed a larger P300 wave amplitude in response to visual stimuli than those without ASD. For MMN, there were no significant amplitude differences between groups
Jutla et al. (2020aa)	Cross-sectional	US	151 ASD 425 CHR-P	ASD: pre-existing diagnosis CHR-P: PQ-BC	9.91 (0.62)	52,33	7	ASD increased PQ-BC distress scores, suggesting ASD as a strong predictor of psychotic-like experiences. ASD also had a strong positive effect on PQ-BC distress score. The effect of ASD was also larger than the positive predictor trauma
Jutla et al. (2020b)	Cross-sectional	US	69 ASD 753 CHR-P	n/a	n/a	n/a	3	Retrosplenial-temporal, cinguloparietal, and cingulo-opercular connectivity most strongly predicted ASD without psychotic-like symptoms, ASD with psychotic-like symptoms, and psychotic-like symptoms without ASD respectively. Within the ABCD cohort, ASD with psychotic-like symptoms, ASD without psychotic-like symptoms, and psychotic-like symptoms without ASD were characterised by distinct connectivity patterns. These results suggest that ASD with psychotic-like symptoms may represent an ASD subtype with distinct neural correlates
Maat et al. (2020)	Cross-sectional	The Netherlands	53 CHR-P 21 ASD 81 HC	ASD: SCQ CHR-P: SIPS	15.4 (2.05)	71,7	7	Traditional computerised assessment of facial affect processing is unlikely to detect early vulnerability markers for psychosis in adolescents with APS. A more autistic-like profile may be characterised by a generalised increase in response latencies, suggesting that the combined presence of autistic and psychotic traits may disproportionately affect cognitive performance. The CHR-P group with ASD generally showed slower responses for affective and non-affective face stimuli than CHR-P participants without ASD and healthy controls
Solomon et al. (2008)	Cross-sectional	US	17 ASD 21 HC	ASD: DSM-IV/ADOS/SCQ CHR-P: KFTDS	12.33 (2.31)	n/a	7	In participants with ASD, illogical thinking was related to aspects of cognitive functioning and to executive control. Loose associations were related to autism communication symptoms and to parent reports of stress and anxiety. When formal thought disorder is present in ASD, it is related to pragmatic language abnormalities
Solomon et al. (2011)	Cross-sectional	US	20 ASD 15 CHR-P 16 FEP 20 HC	ASD: DSM-IV/ADOS/SCQ CHR-P: SIPS	15.24 (2.37)	71,83	7	ASD, CHR-P and FEP share common features of atypical neurodevelopment of language and social function. On the Social domain, ASD scored significantly worse. For Cognition, Motivation, and Mannerisms, each clinical group was significantly more impaired than HC. For Communication, ASD scored worse than all groups. On the structural and pragmatic language domains, individuals with ASD showed significantly greater impairment than all groups, including delayed echolalia and deficits in appreciating irony and sarcasm
Sprong et al. (2008)	Longitudinal	The Netherlands	32 ASD 80 CHR-P 82 HC	ASD: DSM-IV/ADI-R/SCQ CHR-P: SIPS/BSABS-P	15.43 (1.82)	55,15	6	Subjects diagnosed with PDD are at high risk for developing psychosis. The CHR-P group reported higher levels of SIPS Positive and Negative symptoms than the ASD-group. ASD and CHR-P did not differ with regard to schizotypal traits and basic symptoms, as well as disorganised and general prodromal symptoms. ASD females at CHR-P scored higher levels on SIPS Positive, SIPS-General, BSABS-P Perceptual and SPQ-R Positive scales
Wilson et al. (2020)	Cross-sectional	US	21 ASD 0 CHR-P 22 HC	ASD: ADI-R/ADOS/SCQ CHR-P: SIPS	14.83 (1.70)	86	7	A majority of verbal responses (93%) and behavioural responses (89%) to SIPS items were rated as adequate, suggesting that the positive domain items from the SIPS can be used with adolescents with ASD. Regardless of diagnosis, higher rates of response errors were significantly correlated with greater difficulty understanding ambiguous language. Results indicate that adolescents with ASD did not significantly differ from typically developing peers when answering questions about positive psychosis risk symptoms

*AD* autistic disorder, *ADI-R* the Autism Diagnostic Interview-Revised, *ADOS* Autism Diagnostic Observation Schedule, *AS* Asperger Syndrome, *ASD* autism spectrum disorder, *BSABS-P* Bonn scale for the assessment of basic symptoms, *CHR-P* clinical high risk for psychosis, *CAARMS* the comprehensive assessment of at risk mental states, *DSM-IV* Diagnostic and Statistical Manual of Mental Disorders, fourth edition, *FEP* first episode of psychosis, *HC* healthy control, *KFTDS* Kiddie-Formal Thought Disorder rating Scale, *MMN* mismatch negativity, *NOS* Newcastle Ottawa Scale, *PDD* pervasive developmental disorder, *PDD-NOS* pervasive developmental disorder not otherwise specified, *PQ* The Prodromal Questionnaire, *PQ-BC* the Prodromal Questionnaire-Brief Child Version, *SCQ* Social Communication Questionnaire, *SIPS* Structured Interview for Prodromal Syndromes, *SOPS* Scale of Prodromal Symptoms, *SPQ-R* Schizotypal Personality Questionnaire-Revised

The mean age of ASD individuals across the included studies was 11.09 years. Likewise, the mean age of CHR-P individuals within the included studies was 18.23 years. Seven (53.8%) studies included only children and adolescents (Eussen et al., 2014; Jutla et al., 2020a, b; Maat et al., 2020; Solomon et al., 2008; Sprong et al., 2008; Wilson et al., 2020). Moreover, most of the included studies had a higher percentage of males (53.5% of the total sample) (Summary Findings in Table 2).

**Table 2:** Summary findings

	Systematic review findings	Metaanalysis ASD-CHR-P
ASD & CHR-P	– Studies: 13 – N = 16,474 individuals **∙** Mean age range: 8.82–18.53 years **∙** Males: 53.5% – The APS group was the most frequent reported: 100%. 4% met criteria for GRD and 3.1% met criteria for BLIPS. 36.7% met criteria for basic symptoms – ADHD was present in 33.0–52.0%. Anxiety disorders in 14.0–44.0%. ODD in 22.0%. Mood disorders in 8.8%. Tourette’s disorder in 5.0% – ASD individuals at CHR-P showed significantly greater impairment in structural and pragmatic language and social functioning domains – CHR-P with ASD had higher impairment in facial affect recognition and showed slower responses for affective and non-affective face stimuli than those without ASD – ASD individuals at CHR-P had poorer social cognition – ASD at CHR-P showed a larger P300 wave amplitude in response to visual stimuli – Female ASD individuals at CHR-P had more general and positive symptoms on the SIPS – The presence of formal thought disorder in ASD ranged between 16.5 and 60.4% – ASD at CHR-P showed more social anhedonia – The frequency of ASD at CHR-P was reported as being 1.1–39.6% – the occurrence of CHR-P in ASD oscillated between 0 to 78.0% – Conversion rates of ASD at CHR-P range from 15.4 to 18.2% at 2 years of follow-up. ASD status was not associated with differential rates of conversion – The most used psychopharmacological treatment were antipsychotics	– 11.6% (95% CI 2.1–44.2) of CHR-P individuals have an ASD diagnosis – Heterogeneity was significant, (Q = 75.157, I^2^ = 96.008%) – Egger’s test result (0.425) did not reveal significant publication bias, (p = 0.712)
ASD vs CHR-P	– The mean age was 11.09 years for ASD – The mean age was 18.23 years for CHR-P – Among those CHR-P individuals without ASD, 91.3–100% met the APS criteria; 0.8–11.3% met BLIPS criteria and 4.6–11.3% met GRD criteria – ASD individuals without CHR-P had less impairment on social cognition – ASD with psychotic-like symptoms and ASD without psychotic-like symptoms are characterized by distinct connectivity patterns. Retrosplenial-temporal, cinguloparietal, and cingulo-opercular connectivity most strongly predicted ASD without psychotic-like symptoms, ASD with psychotic-like symptoms, and psychotic-like symptoms without ASD respectively – Conversion rate in CHR-P without ASD ranged 11.1–14% at 2 years – Antipsychotics (19.0–50.0% versus 25.0%) and psychostimulants (15.6–48.0% versus 2.5%) were prescribed more frequently in ASD than in CHR-P – Anxiolytics (11.3% vs 5.0%) were prescribed more frequently in CHR-P than in ASD	

*ADHD* attention deficit hyperactivity disorder, *APS* attenuated psychotic symptoms, *ASD* autism spectrum disorder, *BLIPS* brief limited intermittent psychotic symptoms, *BS* basic symptoms, *CHR-P* clinical high risk for psychosis, *GRD* genetic risk and deterioration syndrome, *ODD* oppositional defiant disorder, *SIPS* Structured Interview for Prodromal Syndromes

At baseline, and considering the different CHR-P groups, (APS, BLIPS and GRD), the APS group was the most frequently reported in the CHR-P sample with ASD, reaching up to 100% (Foss-Feig et al., 2019; Maat et al., 2020; Solomon et al., 2011). In addition, 4% met criteria for GRD (Foss-Feig et al., 2019) and 3.1% fulfilled criteria for BLIPS (Sprong et al., 2008). Furthermore, 36.7% ASD individuals met criteria for basic symptoms (Sprong et al., 2008). Among those CHR-P individuals without ASD, 91.3–100% met the APS criteria (Foss-Feig et al., 2019; Maat et al., 2020; Solomon et al., 2011; Sprong et al., 2008), 4.6–11.3% met GRD criteria (Foss-Feig et al., 2019; Maat et al., 2020; Sprong et al., 2008) and 0.8–11.3% met BLIPS criteria (Foss-Feig et al., 2019; Maat et al., 2020; Sprong et al., 2008).

### Clinical Comorbidity

Two studies also reported on the presence of other symptomatic domains in ASD (Eussen et al., 2014; Wilson et al., 2020). Symptoms of anxiety were reported between 14.0 (Wilson et al., 2020) to 44.0% (Eussen et al., 2014). In addition, between 33.0 (Eussen et al., 2014) and 52.0% (Wilson et al., 2020) of those individuals with ASD met criteria for Attention Deficit Hyperactivity Disorder (ADHD). Further, 22.0% were diagnosed with Oppositional Defiant Disorder (ODD), 8.8% with Mood Disorder (Eussen et al., 2014) and 5.0%. with Tourette’s Disorder (Wilson et al., 2020).

### Functioning, Cognition and Quality of Life

Individuals with ASD at CHR-P showed: (i) a significantly greater impairment in structural and pragmatic language, and social functioning domains compared to subjects at CHR-P without ASD (Solomon et al., 2011); (ii) a considerably poorer global functioning than those ASD individuals without CHR-P (Foss-Feig et al., 2018, 2019; Sprong et al., 2008); and (iii) a poorer social cognition than ASD without CHR-P (Foss-Feig et al., 2018, 2019).

In terms of social functioning, CHR-P individuals with ASD had higher impairment in facial affect recognition (Maat et al., 2020) and showed slower responses for affective and non-affective face stimuli than those without ASD (Maat et al., 2020).

With regard to cognitive functioning, the mean IQ in individuals with ASD ranged between 94.4 to 114.0 (Eussen et al., 2014; Foss-Feig et al., 2019; Maat et al., 2020; Solomon et al., 2008, 2011; Sprong et al., 2008; Wilson et al., 2020).

### Neuroimaging & Neurophysiology

Considering neural correlates between both conditions, one study reported on resting-state functional connectivity in youth with co-occurring ASD with prodromal psychosis symptoms, showing that the cingulo-parietal connectivity most strongly predicted ASD with psychotic-like symptoms (Jutla et al., 2020a, b). With regard to electrophysiological findings, ASD individuals at CHR-P showed a larger P300 wave amplitude in response to visual stimuli than CHR-P subjects without ASD (Foss-Feig et al., 2018; Guillory et al., 2018a, b).

### Clinical Characteristics, Prediction of Outcomes and Conversion

In our systematic review, prodromal psychosis symptoms in ASD appeared in young people, (mean age range 8.82–18.53). Interestingly, although individuals with ASD and CHR-P were more frequently male, (ranging from 52.3 to 90.1%), female ASD individuals at CHR-P were somewhat more impaired, obtaining higher scores on the SIPS Positive symptoms (Sprong et al., 2008), the SIPS-General symptoms (Sprong et al., 2008) and the BSABS-P Perceptual disturbances subscales (Sprong et al., 2008).

At baseline, positive psychotic symptoms were common in ASD. The occurrence of formal thought disorder in ASD ranged between 16.5 and 60.4% (Eussen et al., 2014). Likewise, attenuated positive symptoms were common in ASD at follow-up: 37.5% had non-bizarre ideas (Eussen et al., 2014), 40.6% unusual thought content (Eussen et al., 2014), and 43.7% displayed perceptual disturbances (Eussen et al., 2014).

Considering the negative symptoms, ASD individuals at CHR-P showed more social anhedonia than those at CHR-P without ASD (Foss-Feig et al., 2019). Furthermore, schizotypal traits were more common in ASD and CHR-P than in the control group (Maat et al., 2020; Sprong et al., 2008). However, there were no differences regarding schizotypal traits between ASD and CHR-P (Maat et al., 2020; Sprong et al., 2008).

The presence of ASD in CHR-P ranged between 1.1 and 39.6% (Foss-Feig et al., 2018, 2019; Fraser et al., 2008; Guillory et al., 2018a, b; Maat et al., 2020; Solomon et al., 2011). On the other hand, the occurrence of prodromal psychotic symptoms in ASD oscillated between 0 to 78.0% (Jutla et al., 2020a, b; Sprong et al., 2008; Wilson et al., 2020).

**Fig. 2:**
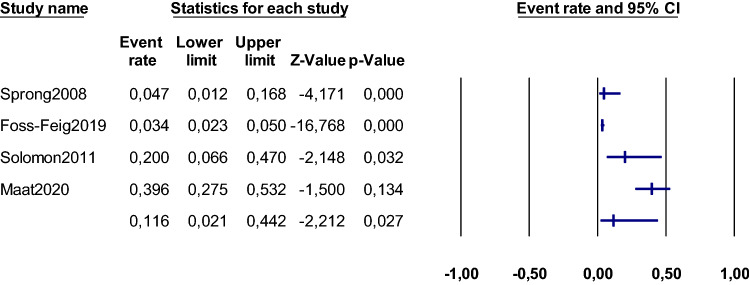
Meta-analysis: ASD in CHR-P. Forest plot

Four studies had data that allowed meta-analyses, comprising 875 individuals at CHR-P (Foss-Feig et al., 2019; Maat et al., 2020; Solomon et al., 2011; Sprong et al., 2008). According to our meta-analysis, 11.6% (95% CI 2.1–44.2) of CHR-P individuals have an ASD diagnosis (Foss-Feig et al., 2019; Maat et al., 2020; Solomon et al., 2011; Sprong et al., 2008) (Fig. 2). Heterogeneity was significant, (Q = 75.157, I^2^ = 96.008%). Additionally, Egger’s test result (0.425) did not reveal significant publication bias (p = 0.712) (eSupplementary Table 5; eSupplementary Fig. 1).

Conversion rates to psychosis of ASD individuals at CHR-P ranged from 15.4 to 18.2% at 2 years of follow-up (Foss-Feig et al., 2018, 2019; Guillory et al., 2018a, b), in comparison with conversion rates in CHR-P without ASD, which ranged 11.1–14.0% at 2 years (Foss-Feig et al., 2018, 2019; Guillory et al., 2018a, b). Further, formal thought disorder in ASD was associated with increased severity of ASD symptoms, but not with conversion to psychosis (Eussen et al., 2014) (eSupplementary Table 6).

### Interventions

The use of psychotropics was more frequent in those with ASD than those with CHR-P, (62.5% vs 45.0% respectively) (Sprong et al., 2008), with a predominance of antipsychotic prescriptions (Sprong et al., 2008). Antipsychotics (19.0–50.0% versus 25.0%) and psychostimulants (15.6–48.0% versus 2.5%) were prescribed more frequently in ASD than in CHR-P (Sprong et al., 2008; Wilson et al., 2020). Conversely, anxiolytics (11.3% versus 5.0%) were prescribed more frequently in CHR-P than in ASD (Sprong et al., 2008; Wilson et al., 2020). Antidepressants were given in 12.5–38.0% individuals with ASD and in 22.5% in individuals at CHR-P (Sprong et al., 2008; Wilson et al., 2020). Response to treatment was not reported. There are no clinical trials in ASD at CHR-P.

### Quality Assessment

The quality of the studies included was 5.31 ± 1.85 and ranged from 3 to 7. The complete results are detailed in eSupplementary Tables 7 and 8.

## Discussion

To our knowledge, this is the first systematic review and meta-analysis to comprehensively evaluated the evidence on the relationship between ASD and CHR-P.

We systematically reviewed 13 studies, focusing on both distinctive and overlapping features between both conditions. We further addressed meta-analytically the presence of ASD in individuals at CHR-P. Our overall sample included a total of 16,474 individuals, despite the fact that the majority of CHR-P studies specifically try to exclude ASD.

Although individuals with ASD with prodromal psychosis may represent a heterogeneous group, its baseline sociodemographic characteristics are now clearer. Typically, these individuals are young, (mean age range 8.82–18.53 years), males (53.5%) who mostly present with APS (up to 100%), and have associated impairments in global functioning, social functioning and social cognition.

Considering the different CHR-P groups, our review showed that up to 100% of ASD individuals at CHR-P fulfilled APS criteria (Foss-Feig et al., 2019; Maat et al., 2020; Solomon et al., 2011), 4.0% met GRD criteria (Foss-Feig et al., 2019) and 3.1% met criteria for BLIPS (Sprong et al., 2008). Likewise, 36.7% met criteria for Basic Symptoms (Sprong et al., 2008). Our review showed that the proportion fulfilling criteria for APS was somewhat higher (100% vs 85%) and lower for BLIPS (3.1% vs 10%) in ASD at CHR-P compared to that reported by Fusar-Poli et al. in CHR-P individuals (Fusar-Poli et al., 2020a, b). This difference may be because the majority of CHR-P studies exclude individuals with ASD.

Furthermore, at presentation, ASD individuals with prodromal psychosis symptoms often had other non-psychotic symptoms. The most commonly reported were ADHD symptoms, present in up to 52% in our review (Wilson et al., 2020), which is somewhat higher (33.0–52.0% vs 28.0%) compared to that reported in prior studies (Lai et al., 2019; Simonoff et al., 2008). Regarding ADHD symptoms, prior literature has reported that attenuated psychotic experiences in ASD are associated with the co-occurrence of attention problems (Gadow, 2012). The second most common was anxiety-related symptoms reported in up to 44% (Eussen et al., 2014), which is no different from that previously described in ASD subjects in some studies, informed in 41.9% (Simonoff et al., 2008). However, this rate was higher than reported by other researchers, where the prevalence of anxiety in ASD was reported in 20.0% (Lai et al., 2019). In our review, the rate of anxiety is much higher compared to previous studies on CHR-P, where anxiety symptoms were present in 15.3% (Fusar-Poli et al., 2014a, b) and 21.3% (Ribolsi et al., 2017) of individuals with attenuated psychosis syndrome. Additionally, 8.8% of individual with ASD with formal thought disorder met criteria for mood disorder (Eussen et al., 2014). This figure is no different from individuals with ASD without CHR-P, where depressive disorder is reported in 1.4% (Simonoff et al., 2008) to 12.9%. (Leyfer et al., 2006). Nevertheless, this rate was much lower than that reported in previous studies in individuals at CHR-P (Fusar-Poli et al., 2014a, b). A recent meta-analysis reported that 40.7% of CHR-P population have comorbid depressive disorders (Fusar-Poli et al., 2014a, b). Individuals at CHR-P are characterized by a high prevalence of depressive and anxiety symptoms in addition to their attenuated psychotic symptoms (Fusar-Poli et al., 2014a, b). These symptoms might reflect essential emotional dysregulation processes and delusional mood in prodromal psychosis (Fusar-Poli et al., 2014a, b). In this line, there is some evidence that emotional disturbances in ASD may mediate psychotic experiences (Solomon et al., 2008). Specifically, formal thought disorder in ASD can be induced by anxiety (Solomon et al., 2008), and higher rates of psychotic experiences in ASD is associated with significant symptoms of anxiety and thought disorder (Sprong et al., 2008).

According to our results, social difficulties are present in ASD and CHR-P, which is in line with prior studies (Addington et al., 2017; Sugranyes et al., 2011b). We have highlighted that ASD individuals at CHR-P had more impaired social functioning (Foss-Feig et al., 2018, 2019; Sprong et al., 2008), and poorer social cognition (Foss-Feig et al., 2018, 2019) than ASD without prodromal psychosis. CHR-P individuals with ASD had higher impairment in facial affect recognition, and showed slower responses for affective and non-affective face stimuli (Maat et al., 2020). This is consistent with the earlier concept that facial recognition and processing in ASD is impaired (Tang et al., 2015). Further, it is undisputed that ASD (Black et al., 2017) and psychotic disorders (Savla et al., 2013) are characterized by deficits in facial emotion processing.

When cognition is considered, various hypotheses have been suggested to explain how neurological functions are altered in ASD and psychotic disorders (Baron-Cohen & Belmonte, 2005; Brüne, 2004; Burns, 2004; Happé & Frith, 2006). A point of consensus in these hypotheses is that cognitive dysfunctions are determined by altered interactions between and within components of the human social brain, including the amygdala, orbitofrontal cortex, anterior cingulate cortex, medial prefrontal cortex, and the mirror-neuron systems, among other neurological structures (Crespi & Badcock, 2008).

Increased psychosis rates in individuals with ASD (Gadow, 2012; Konstantareas & Hewitt, 2001; Selten et al., 2015); autistic traits leading to a higher risk of psychosis (Bevan Jones et al., 2012; Sullivan et al., 2013) molecular genetics findings (Burbach & van der Zwaag, 2009; Guilmatre et al., 2009); neuroimaging findings (Eack et al., 2017; Foss-Feig et al., 2017), and deficits in social cognition (Pinkham et al., 2008; Sugranyes et al., 2011a, b) may indicate a common pathway between ASD and psychosis.

Several overlapping symptoms between ASD and CHR-P have been described. (Table 3). Taking into account cognitive models in computational neuroscience, ASD and psychotic symptoms might result from an imbalance between top-down and bottom-up perceptual processing (van Schalkwyk et al., 2017). From a cognitive neuroscience perspective, there are multiple areas of overlap between ASD and psychotic disorders, and this theoretical overlap might explain the overlapping symptomatology between both conditions (van Schalkwyk et al., 2017), especially in higher-level predictions and social function, manifest as delusional beliefs (van Schalkwyk et al., 2017). Further, from a clinical perspective, psychotic symptoms appear to occur in ASD more frequently than may be expected (Dossetor, 2007; Van Schalkwyk et al., 2015). In this line, a retrospective study of 84 individuals with ASD found that 51% had psychotic symptoms (Kyriakopoulos et al., 2015), suggesting that psychosis might be particularly common in ASD. Additionally, a previous study showed that between 20 and 50% of people with childhood-onset schizophrenia had ASD (Rapoport et al., 2009). Further, ASD traits appear to be prevalent in 9.6–61.0% of individuals with psychosis (Kincaid et al., 2017).

**Table 3:** Symptom overlap between ASD and CHR-P

Symptoms	ASD	CHR-P	Comments
Symptoms present before 3 years (early developmental period)	+++	–	In ASD, symptoms typically appear during pre-school years
Emergence of symptoms in adolescence or young adulthood	–	+++	Not seen in ASD. CHR-P usually appears in 12–35-year-olds individuals
Deficits in social-emotional reciprocity	+++	+	Core ASD symptom. May appear in CHR-P
Deficits in nonverbal communicative behaviours	+++	+	Core ASD symptom. May appear in CHR-P
Deficits in developing, maintaining, and understanding relationships	+++	+	Core ASD symptom. May appear in CHR-P
Stereotyped or repetitive motor movements, use of objects, or speech	+++	++	Core symptom in ASD. May appear in CHR-P
Insistence on sameness, inflexible adherence to routines, or ritualised patterns of behaviour	+++	+	Core symptom in ASD. Rituals may occasionally appear in CHR-P
Highly restricted, fixated interests	+++	+	Core symptom in ASD. Uncommon in CHR-P
Hyper or hypo-reactivity to sensory input or unusual interest in sensory aspects of environment	+++	–	Core symptom in ASD. Does not typically appear in CHR-P
Unusual thought content	+	+++	Core symptom in CHR-P. May appear in ASD associated with a circumscribed intense interest
Thought interference	–	+++	Core symptom in CHR-P. Not typical in ASD
Thought perseveration	++	+++	Core symptom in CHR-P. May appear in ASD
Thought pressure	–	++	Frequent in CHR-P. Not typical in ASD
Thought blockages	–	++	Frequent in CHR-P. Not typical in ASD
Suspiciousness	+	+++	Core symptom in CHR-P. May appear in ASD, particularly in those who misconstrue social cues
Unstable ideas of reference	–	+++	Frequent in CHR-P. Not typical in ASD
Perceptual abnormalities	+	+++	Core symptom in CHR-P. May appear in ASD as part of ‘imaginary friends’ since early life and not a new phenomenon
Visual perception disturbances	+	++	Core symptom in CHR-P. May appear in ASD due to sensory issues
Acoustic perception disturbances	+	+++	Core symptom in CHR-P. May appear in ASD due to sensory hyperresponsivity but is not a new-onset phenomenon
Decreased ability to discriminate between ideas, perception, fantasy and true memories	+	++	Frequent in CHR-P. May appear in ASD
Derealisation	–	++	Frequent in CHR-P. Not typical in ASD
Disorganised communication	+	+++	Core symptom in CHR-P. May appear in ASD
Disturbance of expressive speech	++	++	Core symptom in CHR-P. May appear in ASD
Disturbance of receptive speech	+++	+++	Core symptom in CHR-P and ASD
Grandiose ideas	–	+++	Core symptom in CHR-P. Not typically present in ASD
Disturbances of abstract thinking	+++	++	Core symptom in ASD. May appear in CHR-P
Inability to divide attention	++	++	May appear in ASD and CHR-P
Captivation of attention by details of the visual field	+++	++	Core symptom in ASD. May appear in CHR-P
Mannerism	+++	+	Core symptom in ASD. May appear in CHR-P
Stereotypy	+++	+	Core symptom in ASD. May appear in CHR-P
Agitation not influenced by external stimuli	++	++	Frequently reported in ASD and CHR-P

*ASD* autism spectrum disorder, *CHR-P* clinical high risk for psychosis

To refine detection and prognosis at the individual level, future research may contemplate specific risk factors (e.g., sex, trauma and living status), cognitive functioning (e.g., processing speed and verbal and visual memory) and biomarkers (e.g., neuroimaging data).

Considering neuroimaging findings, our review showed that ASD with psychotic-like symptoms and ASD without psychotic-like symptoms are characterized by distinct connectivity patterns (Jutla et al., 2020a, b), which is in line with prior studies from the literature, where stimulus-specific differences between both conditions have been reported (Sugranyes et al., 2011a, b).

Our findings suggest that individuals at CHR-P with ASD were younger than CHR-P without autism. The differentiating feature is that the symptoms in CHR-P are new-onset, usually during late adolescence, unlike ASD, which begins in early pre-school years. Furthermore, in most services and studies that include CHR-P population, the age of the individuals included typically ranges between 16 and 35 years (Fusar-Poli et al., 2013b, 2020b). Our findings also showed that ASD and CHR-P appear more frequently in males; nonetheless, female sex was associated with more general and positive symptoms on the SIPS in ASD individuals at CHR-P compared to males (Sprong et al., 2008). This is in line with evidence from a prior study on subclinical psychotic symptoms in the general population, which showed that females individuals had higher rates of positive psychotic experiences (Maric et al., 2003).

The findings also indicate that prodromal psychotic symptoms are common in ASD, with perceptual disturbances, unusual thought content and non-bizarre ideas being high (Eussen et al., 2014; Solomon et al., 2008). Further, 16.5% displayed loose associations and 60.4% had illogical thinking (Eussen et al., 2014). Additionally, loose associations were related to autism communication deficits and anxiety (Solomon et al., 2008). When features of formal thought disorder are present in ASD, they are not necessarily co-morbid psychotic disorder, but likely are evidence of symptomatic overlap with the pragmatic language abnormalities inherent in autism. The increase in the identification of these psychotic experiences in ASD may be due to the increased interest in early detection, and because the symptoms may appear in both conditions.

Our meta-analysis showed that 11.6% (95% CI 2.1–44.2) of CHR-P individuals have an ASD diagnosis, which is in keeping with the results from the review. Our findings showed that the presence of ASD in CHR-P ranged between 1.1 and 39.6% (Foss-Feig et al., 2018, 2019; Fraser et al., 2008; Guillory et al., 2018a, b; Maat et al., 2020; Solomon et al., 2011). Interestingly, the occurrence of CHR-P in ASD oscillated between 0 to 78.0% (Jutla et al., 2020a, b; Sprong et al., 2008; Wilson et al., 2020). This discrepancy may be due to the heterogeneity of the samples, with different sample sizes, as well as the use of different diagnostic measures to define CHR-P status. Additionally, a sample included individuals who did not meet strict criteria for autism but did fulfil the criteria for the Multiple Complex Developmental Disorder (MCDD), a distinct group within the autistic spectrum based on symptomatology (Cohen et al., 1986, 1994), with more psychotic features, constituting a population at risk, with greater vulnerability to develop psychosis (Jansen et al., 2000). Furthermore, MCDD is characterized by early childhood-onset emotional dysregulation with high levels of anxiety, aggressiveness and thought disorder, with a remarkably elevated risk of psychosis (Cohen et al., 1994; Van Engeland & Van der Gaag, 1994) (eSupplementary Table 9).

Further, we also found that conversion rates of ASD individuals at CHR-P ranged from 15.4 to 18.2% at 2 years of follow-up, which was not different from CHR-P without ASD, (11.1–14.0% at 2 years) (Eussen et al., 2014; Foss-Feig et al., 2018, 2019; Guillory et al., 2018a, b). ASD status was not associated with differential rates of conversion in comparison with CHR-P individuals without ASD. Besides, our rate was slightly lower than reported by other researchers in CHR-P population, where the transition risk was 20% at 2 years (Salazar De Pablo et al., 2020), 22–23% at 3 years (Fusar-Poli et al., 2020a; Salazar De Pablo et al., 2020), and 19.6% at 6 years of follow-up (Ziermans et al., 2014).

Psychopharmacological treatment, especially antipsychotics, are used more frequently in subjects with ASD than in CHR-P (Sprong et al., 2008). In our review, the proportion of ASD treated with antipsychotics was somewhat higher, (19.0–50.0% vs 7.0–34.0%), compared to prior studies (Coury et al., 2012; Downs et al., 2016; Eussen et al., 2014; Murray et al., 2014) The increased use of antipsychotics may be related to its use in the treatment of behavioural symptoms in ASD (Alfageh et al., 2019; Owen et al., 2009; Shea et al., 2004). Moreover, a recent meta-analysis showed that there is little evidence to favour the use of antipsychotics to improve attenuated psychotic symptoms in CHR-P (Davies et al., 2018). Likewise, according to a previous meta-analysis, there is no evidence to support the superior efficacy of any intervention over another to reduce attenuated positive psychotic symptoms.

## Limitations

Limitations include the small number of studies evaluated and the heterogeneity of diagnostic criteria used in these studies. Firstly, most of the studies on CHR-P specifically exclude patients with ASD, therefore we have fewer studies to include into this analysis. Also, due to the lack of sufficient data on both CHR-P and ASD in publications, we could only use four studies in our meta-analysis to evaluate the presence of ASD in CHR-P. Secondly, no gold-standard measures have been used to define ASD in these studies. Likewise, there is no gold-standard measure to define CHR-P status. Nevertheless, the four studies included in the metanalysis defined CHR-P state using the Structured Interview for Prodromal Syndromes (SIPS), which is a validated measure to describe CHR-P (Fusar-Poli et al., 2016). Thirdly, due to the lack of data in publications, we were not able to meta-analyse the presence of CHR-P in ASD. And fourth, the scarce available data did not allow us to do meta-regression analyses to examine the relationship of characteristics such as clinical, psychopathological, therapeutic, cognitive and neurobiological aspects within both conditions. Nevertheless, the total number of participants included in the current metanalysis is sizeable and the results are significant with precise 95% CIs.

Longitudinal studies are required to address the overlap between ASD and CHR-P. Special consideration may need to be given to how to assess prodromal psychotic experiences in individuals with ASD. Lifelong symptoms with pre-school onset should be differentiated from recent-onset symptoms. Symptom ratings for CHR-P should ensure that only symptoms that are of recent onset are used during CHR-P evaluation to avoid diagnostic overshadowing. Longitudinal studies that measure anxiety, low mood, stressful life events and treatment response in CHR-P, with and without ASD, will further our understanding.

## Conclusion

Features of prodromal psychosis are present in individuals with ASD. They are more common in males, but females may display more severe symptoms. There is evidence of psychopathological overlap between both conditions, which may hinder the diagnostic process and treatment. Individuals with ASD at CHR-P have significantly poorer global functioning and poorer social cognition than ASD without CHR-P. There are no differences in comorbidity in ASD with or without CHR-P. The presence of ASD is not associated with conversion to psychosis. Prompt detection, assessment, and intervention in this population have the potential to maximise the benefits of early interventions.

## Supplementary Information

Below is the link to the electronic supplementary material.

Supplementary file1 (DOCX 108 kb)
