# Supplementary material for: Autism Spectrum Disorder and Clinical High Risk for Psychosis: A Systematic Review and Meta-analysis
Source: J Autism Dev Disord. 2021 May 15;52(4):1568–86. doi: 10.1007/s10803-021-05046-0 (PMC8938385; doi:10.1007/s10803-021-05046-0)
Supplement: Supplementary file 1 — Supplementary file1 (DOCX 108 kb) [file 10803_2021_5046_MOESM1_ESM.docx]

**SUPPLEMENTARY MATERIAL**

- **eTable 1.** Diagnostic Criteria for Clinical High-Risk for Psychosis (CHR-P).
- **eTable 2.** Diagnostic criteria for Basic Symptoms.
- **eTable 3.** MOOSE checklist.
- **eMethods 1**: Assessment measures of Autism Spectrum Disorder (ASD).
- **eMethods 2**: Assessment measures of CHR-P.
- **eTable 4:** Risk of bias (quality) assessment using the modified Newcastle Ottawa Scale for cross-sectional and longitudinal studies.
- **eTable 5:** Meta-analytical results. Rate of ASD in CHR-P, heterogeneity and publication bias.
- **eFigure 1:** Funnel Plot results for ASD in CHR-P.
- **eTable 6:** Proportion of CHR-P and Basic Symptoms in ASD with conversion rates in our review.
- **eTable 7:** Risk of bias (quality assessment) using modified Newcastle Ottawa Scale for longitudinal studies.
- **eTable 8:** Risk of bias (quality assessment) using modified Newcastle Ottawa Scale for cross-sectional studies.
- **eTable 9:** Diagnostic criteria for Multiple Complex Developmental Disorder (MCDD).

This supplementary material has been provided by the authors to give readers additional information about their work.

**eTable 1. Diagnostic Criteria for Clinical High-Risk for Psychosis (CHR-P) according to SIPS.**

| CHR-P  (Fusar-Poli et al., 2016; McGlashan, T., Walsh, B., & Woods, 2019) | Attenuated psychotic symptoms | Brief limited intermittent psychotic symptoms | Genetic risk and deterioration syndrome |
| --- | --- | --- | --- |
| Inclusion criteria | SIPS positive symptom scales: (P1) unusual thought content, (P2) suspiciousness, (P3) grandiose ideas, (P4) perceptual abnormalities, and (P5) disorganized communication, with at least 1 of these symptoms rated 3, 4, or 5, indicating clinically significant disturbance below a psychotic level of intensity. | SIPS positive symptom scales: (P1) unusual thought content, (P2) suspiciousness, (P3) grandiose ideas, (P4) perceptual abnormalities, and (P5) disorganized communication, with at least 1 of these symptoms rated, 6 indicating clinically severe disturbance with psychotic features. | The patient meets criteria for Schizotypal Personality Disorder OR has a first-degree relative with a psychotic disorder. |
| Frequency | Symptoms ever been present at an average frequency of at least once per week over a month. | Symptoms ever been present at least several minutes per day, at least one month. |  |
| Onset | Symptoms should have begun within the past year OR currently rate one or more scale points higher compared to 12 months before. Symptoms that occurred over the past month only are rated. | Symptoms should have reached a psychotic level of intensity in the previous 3 months. |  |
| Level of functioning | No social/occupational dysfunction requirement. | No social/occupational dysfunction requirement. | 30% drop in GAF score over the last month as compared to 12 months before. |
| Duration |  | Up to 3 months. |  |

**eTable 2. Diagnostic criteria for Basic Symptoms.**

| Basic Symptoms  (Schultze-Lutter et al., 2012; Schultze-Lutter & Theodoridou, 2017) | COPER | COGDIS |
| --- | --- | --- |
| BS are subtle, subjectively experienced disturbances in mental processes including thinking, attention, perception, speech, stress tolerance, and affect. | Presence of at least any one of the following ten basic symptoms, with at least weekly occurrence within the last three months and first occurrence at least 12 months ago:   - Thought interference. - Thought perseveration. - Thought pressure. - Thought blockages. - Disturbance of receptive speech. - Decreased ability to discriminate between ideas and perception, fantasy and true memories. - Unstable ideas of reference. - Derealisation. - Visual perception disturbances. - Acoustic perception disturbances. | Presence of at least any two of the following nine basic symptoms, with at least weekly occurrence within the last three months:   - Inability to divide attention. - Thought interference. - Thought pressure. - Thought blockages. - Disturbance of receptive speech. - Disturbance of expressive speech. - Unstable ideas of reference. - Disturbances of abstract thinking. - Captivation of attention by details of the visual field. |
| BS are regarded as an immediate symptomatic expression of the neurobiological processes underlying psychosis and the earliest form of self‐experienced symptoms. |  |  |
| Two criteria for the identification of basic symptoms:   - Cognitive‐Perceptive Basic Symptoms (COPER). - Cognitive Disturbances (COGDIS). |  |  |

**eTable 3: MOOSE checklist. (Stroup et al., 2000)**

| **Criteria** | | **Brief description of how the criteria were handled in the meta-analysis** |
| --- | --- | --- |
| **Reporting of background should include** | |  |
| √ | Problem definition | No meta-analysis has evaluated the presence of ASD in CHR-P. |
|  | Hypothesis statement |  |
| √ | Description of study outcomes | Studies were described in the supplementary table. |
|  | Type of exposure or intervention used |  |
| √ | Type of study designs used | Both cross sectional and longitudinal studies were selected |
| √ | Study population | Subjects with ASD, CHR-P and catatonia |
| **Reporting of search strategy should include** | |  |
| √ | Qualifications of searchers | The credentials of the investigators are indicated in the author list and in the acknowledgements. |
| √ | Search strategy, including time period included in the synthesis and keywords | We performed a multi-step literature search using keywords described in methods section: until 21st April 2020. |
| √ | Databases and registries searched | Web of Science database (Web of Science Core Collection, BIOSIS Citation Index, KCI-Korean Journal Database, MEDLINE, Russian Science Citation Index, and SciELO Citation Index) and grey literature. |
| √ | Use of hand searching | References of systematic reviews or meta-analyses that were screened during literature search and the references from the included studies were manually searched. |
| √ | List of citations located and those excluded, including justifications | Details of the literature search process are outlined in the results section and PRISMA flowchart. |
| √ | Method of addressing articles published in languages other than English | Only articles in English language were selected. |
| √ | Method of handling abstracts and unpublished studies | Original individual studies were included. Reviews, clinical cases and study protocols were excluded. |
| √ | Description of any contact with authors | We did not contact authors. |
| **Reporting of methods should include** | |  |
| √ | Description of relevance or appropriateness of studies assembled for assessing the hypothesis to be tested | Detailed inclusion and exclusion criteria are described in the methods section. |
| √ | Rationale for the selection and coding of data | Data extracted from each of the studies are relevant to the population characteristics, study design and studies outcomes. |
| √ | Assessment of confounding | We did not investigate confounding factors as stated in the limitations section. |
| √ | Assessment of study quality, including blinding of quality assessors; stratification or regression on possible predictors of study results | We evaluated the quality using Mixed Methods Appraisal tool |
| √ | Assessment of heterogeneity | Heterogeneity was assessed with the I^2^ index. |
| √ | Description of statistical methods in sufficient detail to be replicated | A random-effects meta-analysis was used. Heterogeneity among study point estimates was assessed using Q statistics. The proportion of the total variability in the effect size estimates was evaluated with the I^2^ index. |
| √ | Provision of appropriate tables and graphics | We included the PRISMA flow-chart to describe the literature search and its results. |
| **Reporting of results should include** | |  |
| √ | Table summarizing individual study estimates and overall estimate | We reported this in the results. |
| √ | Table giving descriptive information for each study included | We have presented descriptive information for each study in the tables and as supplementary material. |
| √ | Results of sensitivity testing | We did not conduct subgroup analyses |
| √ | Indication of statistical uncertainty of findings | We reported this in the results section |
| **Reporting of discussion should include** | |  |
| √ | Quantitative assessment of bias | Publication biases was assessed using a modified version of the Newcastle-Ottawa Scale was used for cross-sectional and longitudinal studies. Scores ranged from 0 to 8 |
| √ | Justification for exclusion | We excluded studies based on the rationale of the meta-analysis |
| √ | Assessment of quality of included studies | The quality of the studies was assessed and reported. |
| **Reporting of conclusions should include** | |  |
| √ | Consideration of alternative explanations for observed results | We have addressed this point in the discussion section |
| √ | Generalization of the conclusions | We have addressed this point in the discussion section. |
| √ | Guidelines for future research | We have addressed this point in the discussion section |
| √ | Disclosure of funding source | Funding source was specified. |

**eMethods 1**: **Assessment measures of Autism Spectrum Disorder.**

Autism Spectrum Disorders are early onset conditions categorized by persistent deficits in social communication, and restricted and repetitive patterns of behaviour.(American Psychiatric Association, 2013) After several revisions, according to the fifth edition of the Diagnostic and Statistical Manual of Mental Disorders, (DSM-5), Autism Spectrum Disorders, (hereafter ASD), are included under neurodevelopmental disorders and encompass both Autism Spectrum Disorders and Pervasive Developmental Disorders.(American Psychiatric Association, 2013) In our review, to support the diagnosis, nine studies used the DSM-IV criteria.(Eussen et al., 2014; J. H. Foss-Feig et al., 2019; J. Foss-Feig et al., 2018; Fraser et al., 2008; Guillory et al., 2018a, 2018b; Solomon et al., 2011; Solomon, Ozonoff, Carter, & Caplan, 2008; Sprong et al., 2008) Other diagnosis tools to characterize ASD were also considered in the included studies, such as the Autism Diagnostic Observation Schedule-Generic (ADOS-G);(Eussen et al., 2014; Solomon et al., 2011, 2008; Wilson et al., 2020) the Autism Diagnostic Interview-Revised (ADI-R),(Sprong et al., 2008; Wilson et al., 2020) and the Social Communication Questionnaire (SCQ).(Solomon et al., 2011, 2008; Sprong et al., 2008; Wilson et al., 2020)

The ADI-R (Lord, Rutter, & Le Couteur, 1994) is a semi-structured interview applied by trained examiners to caregivers who collect developmental data about the patient's first years of life on a variety of conducts and skills. It comprises 93 items and 153 ratings and is organized in six sections: early development; acquisition and loss of language/other skills; language and communication functioning; social development and play; interests and behaviours, and general behaviours. The standard algorithms are organized in four sections consistent with the diagnostic definition of autism in the DSM-IV and ICD-10.

The ADOS-Generic (Lord et al., 2000) is a semi-structured assessment of communication, social interaction and play, which can be used to evaluate children or adults with limited or no language, and also those who are verbally fluent. It consists of four modules that are administered according to the verbal level of the individual. Each module contains standard activities that allow the examiner to observe behaviours consistent with a diagnosis of ASD or other pervasive developmental disorders.

The SCQ (Berument, Rutter, Lord, Pickles, & Bailey, 1999) is a 40-item parent-report questionnaire designed to address the severity of impairments in social skills, communication, and repetitive behaviour domains seen in ASD. The SCQ has Lifetime and Current versions. Each item is scored using a dichotomous response format, with a value of 1 indicating the presence of atypicality and a value of zero the absence of atypicality. The first item is not scored, however determines whether the individual is verbal. For verbal children, the range of possible scores is between 0 and 39, and for nonverbal children it is between 0 and 33. A score of 15 has been reported as an optimal cut-off for differentiating between individuals with and without ASD.

**eMethods 2**: **Assessment measures of Clinical High-Risk for Psychosis.**

In our systematic review, all included studies reported on general characteristics of ASD and CHR-P state. Considering the measures to assess the CHR-P status, eight studies used the Structured Interview for Psychosis-Risk Syndromes (SIPS);(J. H. Foss-Feig et al., 2019; J. Foss-Feig et al., 2018; Guillory et al., 2018a, 2018b; Maat, Therman, Swaab, & Ziermans, 2020; Solomon et al., 2011; Sprong et al., 2008; Wilson et al., 2020) four used the Scale of Prodromal Symptoms (SOPS);(J. H. Foss-Feig et al., 2019; J. Foss-Feig et al., 2018; Guillory et al., 2018a, 2018b) one study the Comprehensive Assessment of At Risk Mental States (CAARMS);(Eussen et al., 2014) one used the Prodromal Questionnaire (PQ);(Eussen et al., 2014) two the Prodromal Questionnaire-Brief Child version (PQ-BC);(Jutla, Donohue, Veenstra-Vanderweele, & Foss-Feig, 2020; Jutla, Donohue, Veenstra-VanderWeele, & Foss-Feig, 2020) two the Kiddie-Formal Thought Disorder Rating Scale (KFTDS),(Eussen et al., 2014; Solomon et al., 2008) and one study the Bonn Scale for the Assessment of Basic Symptoms-Prediction List (BSABS-P).(Sprong et al., 2008)

The BSABS-P (Gross, G., Huber, G., Klosterkötter, J., & Linz, 1987) is used to assess 21 self-experienced cognitive, perceptual and motor disturbances found to be predictive for a transition into psychosis. Each basic symptom is given a score of 0 to 6 according to maximum frequency of occurrence during the preceding 3 months as the guiding criterion.

The CAARMS (Yung et al., 2005) is a semi structured interview schedule conducted to determine the presence, severity, frequency, distress and type of CHR symptoms, and includes the following subscales: disorders of thought content (e.g. delusional mood, overvalued ideas and delusions); perceptual abnormalities (e.g. distortions, illusions and hallucinations); conceptual disorganization (e.g. subjectively experienced difficulties with forming thoughts and objective assessment of formal thought disorder); motor changes (e.g. subjectively experienced difficulties with movement and objective signs of catatonia); concentration and attention (measuring both the subjective experience and objective rating); emotion and affect (evaluating subjective sense of change in emotions and objective rating of blunting of affect); subjectively impaired energy, and impaired tolerance to normal stress. Scores for each subscale range from 0 to 6. This instrument classifies individuals in different groups, (meeting CHR criteria, psychosis, or not at risk), according to the severity and frequency of subclinical symptoms. Criteria for CHR are established according only to the four positive symptoms sub-scale (unusual thought content, non-bizarre ideas, perceptual abnormalities and disorganized speech).

The K-FTDS (Caplan, Guthrie, Fish, Tanguay, & David-Lando, 1989) assesses four symptoms of formal thought disorder, based on DSM-III criteria: illogical thinking, loose associations, incoherence, and poverty of content of speech The KFTDS is considered as a reliable measure of Formal thought disorder in children aged 7–18 years.

The PQ (Loewy, R. L., Bearden, C. E., Johnson, J. K., Raine, A., & Cannon, n.d.) assesses the presence and the severity of prodromal symptoms of psychosis and serves to identify young people, with a minimum age of 12 years, at CHR-P in an early stage. The PQ sums up 92 true or false statements about symptoms, which may manifest in the prodromal phase of psychosis. This questionnaire contains a positive scale, measuring symptoms like ideas of reference, delusional ideas and perceptual illusions, and a negative scale, measuring symptoms like decline in social functioning, passivity and withdrawn behaviour. A threshold of 18 on the PQ total score (positive and negative symptoms of psychosis) predicted CHR status with 90 % sensitivity and 38 % specificity.

The PQ-BC (Karcher et al., 2018) is a 21-item screening questionnaire for psychotic-like experiences in adolescents and adults. Each PQ-B item asks about the presence of a psychotic-like experience and then has the respondent rate, on a five-point scale, how much distress the symptom causes if present. To ensure that children understand the questions posed, these items are administered as an interview rather than a questionnaire, with the distress scale paired with a visual response analogue. The PQ-BC is scored by deriving two indices. For the “total” score, ranging from 0 to 21, one point is assigned per symptom endorsed. For the “distress” score, ranging from 0 to 105, 1 to 5 points are assigned per symptom endorsed, based on a distress rating, where 1 indicates “no distress” and 5 “severe distress”.

The SIPS is a diagnostic semi-structured interview, and is complemented by a severity scale, the Scale Of Prodromal Symptoms (SOPS). (McGlashan TWB, 2010; Miller et al., 2002) The two instruments are used to define and diagnose individuals who may be at CHR-P. The goals of both SIPS and SOPS are to provide a systematic measure of the presence/absence of prodromal states, to measure the severity of prodromal symptoms cross-sectionally and longitudinally, and to define an operational threshold for psychosis. The SIPS is composed of 19 items, (five positive symptoms, six negative symptoms, four disorganization symptoms, and four general symptoms); each is given a score of 1 to 6 according to defined criteria. A score between 3 and 5 on the positive symptoms indicates attenuated psychotic symptoms and a score of 6 indicates a psychotic state.

**eTable 4: Risk of bias (quality) assessment using the modified Newcastle Ottawa Scale (NOS) for cross-sectional and longitudinal studies.**

| **Criteria** | **Maximum Score** |
| --- | --- |
| ***Cross-Sectional Studies*** | |
| Sample representative of target sample (e.g., all eligible or random sample)? | 2 |
| Sample size justified and satisfactory? | 1 |
| Non-response rate is defined, satisfactory, and characteristics of responders/non-responders compared? | 1 |
| Ascertainment of exposure is valid and/or well-described? | 1 |
| Assessment of outcome with robust tool and/or record linkage? | 2 |
| Outcome per group reported appropriately? | 1 |
| ***Cohort Studies*** | |
| Representativeness of exposed cohort (e.g. total population or random sample, selected group) | 1 |
| Method used to ascertain exposure is robust? | 1 |
| Exposed and unexposed are matched or adjustment for confounding factors? | 2 |
| Assessment of outcome was blind to exposure status or used record linkage, were robust tools used? | 2 |
| Follow-up period was sufficiently long for outcomes to occur? | 1 |
| Loss to follow-up rate is reported, low (<30%), and same in exposed and non-exposed? | 1 |

**eTable 5. Meta-analytical results. Rate of ASD in CHR-P, heterogeneity and publication bias.**

| **N studies** | **Total** **sample** | **%** | **95%CI** | **Z** | **P value** | **Q** | **df** | **I2** | **P** | **Egger test** | **p** |
| --- | --- | --- | --- | --- | --- | --- | --- | --- | --- | --- | --- |
| 4 | 875 | 11.6 | 2.1-44.2 | -2.212 | 0.027 | 75.157 | 3 | 96.008 | 0.000 | 0.425 | 0.712 |

**eFigure 1: Funnel Plot results for ASD in CHR-P.**

**eTable 6. Proportion of CHR-P and BS in ASD with conversion rates in our review.**

|  | APS | BLIPS | GRD | BS | Conversion rates |
| --- | --- | --- | --- | --- | --- |
| CHR-P/ASD+ | 100% | 3.1% | 4.0% | 36.7% | 15.4-18.2% at 2 years |
| CHR-P/ASD- | 91.3-100.0% | 0.8-11.3% | 4.6-11.3% | n/a | 11.1-14.0% at 2 years |

^ASD: Autism Spectrum Disorder; APS: Attenuated psychotic symptoms; BLIPS: Brief limited intermittent psychotic symptoms; BS: Basic Symptoms; CHR-P: Clinical High Risk for Psychosis; GRD: Genetic risk and deterioration syndrome; CHR-P&ASD: individuals at CHR-P with co-morbid ASD; CHR/ASD-: individuals at CHR-P without co-morbid ASD^

**eTable 7: Risk of bias (quality assessment) using modified Newcastle Ottawa Scale for longitudinal studies.**

| **Study** | **Sample Representativeness** | **Exposure method** | **Groups match and adjustment** | **Assessment of outcome** | **Follow-up period** | **Losses to follow-up** | **Total score** |
| --- | --- | --- | --- | --- | --- | --- | --- |
| Eussem 2014 (Eussen et al., 2014) | 1 | 1 | 2 | 2 | 1 | 0 | 7 |
| Foss-Feig 2018 (J. Foss-Feig et al., 2018) | 1 | 1 | 0 | 0 | 1 | 0 | 3 |
| Foss-Feig 2019 (J. H. Foss-Feig et al., 2019) | 1 | 1 | 2 | 1 | 1 | 0 | 6 |
| Guillory 2018 (Guillory et al., 2018b) | 1 | 1 | 0 | 0 | 1 | 0 | 3 |
| Guillory 2018 (Guillory et al., 2018a) | 1 | 1 | 0 | 0 | 1 | 0 | 3 |
| Sprong 2008 (Sprong et al., 2008) | 1 | 1 | 2 | 1 | 1 | 0 | 6 |

**eTable 8: Risk of bias (quality assessment) using modified Newcastle Ottawa Scale for cross-sectional studies.**

| **Study** | **Sample Representativeness** | **Sample Size** | **Characteristics responders/non responders** | **Ascertainment of exposure** | **Assessment of outcome** | **Outcome reported** | **Total**  **score** |
| --- | --- | --- | --- | --- | --- | --- | --- |
| Fraser 2008 (Fraser et al., 2008) | 1 | 0 | 0 | 0 | 1 | 1 | 3 |
| Jutla 2020 (Jutla, Donohue, Veenstra-VanderWeele, et al., 2020) | 2 | 1 | 1 | 1 | 1 | 1 | 7 |
| Jutla 2020b (Jutla, Donohue, Veenstra-Vanderweele, et al., 2020) | 1 | 1 | 1 | 0 | 0 | 0 | 3 |
| Maat 2020 (Maat et al., 2020) | 1 | 1 | 1 | 1 | 2 | 1 | 7 |
| Solomon2008 (Solomon et al., 2008) | 1 | 1 | 1 | 1 | 2 | 1 | 7 |
| Solomon 2011 (Solomon et al., 2011) | 1 | 1 | 1 | 1 | 2 | 1 | 7 |
| Wilson 2020 (Wilson et al., 2020) | 1 | 1 | 1 | 1 | 2 | 1 | 7 |

**eTable 9. Diagnostic criteria for Multiple Complex Developmental Disorder (MCDD).**

| MCDD  (Buitelaar & Van Der Gaag, 1998; Cohen DJ, Towbin KE, Mayes L, 1994) |  |
| --- | --- |
| 1. Impaired regulation of affective state and anxieties | - 1. Unusual or peculiar fears and phobias or frequent idiosyncratic or bizarre anxiety reactions.   2. Recurrent panic episodes or flooding with anxiety.   3. Episodes of behavioural disorganisation punctuated by markedly immature, primitive or violent behaviours. |
| 1. Impaired social behaviour | 1. Social disinterest, detachment, avoidance or withdrawal despite evident competence. 2. Markedly disturbed and/or ambivalent attachments. |
| 1. The presence of thought disorder | - 1. Irrationality, magical thinking, sudden intrusions on normal thought process, bizarre ideas, neologism or repetition of nonsense words.   2. Perplexity and easy confusability.   3. Overvalued ideas including fantasies of omnipotence, paranoid preoccupations, over-engagement with fantasy figures, referential ideation |
| A diagnosis of MCDD can be made if an individual meets total of five (or more) criteria from (1), (2), and (3), with at least one item from (1), one item from (2) and one item from (3). | |

**REFERENCES**

American Psychiatric Association. (2013). *American Psychiatric Association, 2013. Diagnostic and statistical manual of mental disorders (5th ed.)*. *American Journal of Psychiatry*. https://doi.org/10.1176/appi.books.9780890425596.744053

Berument, S. K., Rutter, M., Lord, C., Pickles, A., & Bailey, A. (1999). Autism screening questionnaire: Diagnostic validity. *British Journal of Psychiatry*. https://doi.org/10.1192/bjp.175.5.444

Buitelaar, J. K., & Van Der Gaag, R. J. (1998). Diagnostic Rules for Children with PDD‐NOS and Multiple Complex Developmental Disorder. *Journal of Child Psychology and Psychiatry*. https://doi.org/10.1111/1469-7610.00391

Caplan, R., Guthrie, D., Fish, B., Tanguay, P. E., & David-Lando, G. (1989). The Kiddie Formal Thought Disorder Rating Scale: Clinical Assessment, Reliability, and Validity. *Journal of the American Academy of Child and Adolescent Psychiatry*. https://doi.org/10.1097/00004583-198905000-00018

Cohen DJ, Towbin KE, Mayes L, V. F. (1994). Developmental psychopathology of multiplex developmental disorder. In H. H. (eds). Friedman SL (Ed.), *Developmental Follow-up: Concepts, Genres, Domains, and Methods.* San Diego: Academic Press.

Eussen, M. L. J. M., de Bruin, E. I., Van Gool, A. R., Louwerse, A., van der Ende, J., Verheij, F., … Greaves-Lord, K. (2014). Formal thought disorder in autism spectrum disorder predicts future symptom severity, but not psychosis prodrome. *European Child and Adolescent Psychiatry*. https://doi.org/10.1007/s00787-014-0552-9

Foss-Feig, J. H., Velthorst, E., Smith, L., Reichenberg, A., Addington, J., Cadenhead, K. S., … Bearden, C. E. (2019). Clinical Profiles and Conversion Rates Among Young Individuals With Autism Spectrum Disorder Who Present to Clinical High Risk for Psychosis Services. *Journal of the American Academy of Child and Adolescent Psychiatry*. https://doi.org/10.1016/j.jaac.2018.09.446

Foss-Feig, J., Velthorst, E., Guillory, S., Hamilton, H., Roach, B., Bachman, P., … Mathalon, D. (2018). Architecture Of Psychosis Symptoms And Neural Predictors Of Conversion Among Clinical High Risk Individuals With Autism Spectrum Disorder. *Schizophrenia Bulletin*. https://doi.org/10.1093/schbul/sby017.649

Fraser, R., Thompson, A., Allott, K., Luxmoore, M., Woodhead, G., & Cotton, S. (2008). Prevalence of autism spectrum disorders in ultra high risk for psychosis and first episode psychosis cohorts. *Early Intervention in Psychiatry*, *2*, A44–A44.

Fusar-Poli, P., Cappucciati, M., Rutigliano, G., Lee, T. Y., Beverly, Q., Bonoldi, I., … McGuire, P. (2016). Towards a Standard Psychometric Diagnostic Interview for Subjects at Ultra High Risk of Psychosis: CAARMS versus SIPS. *Psychiatry Journal*. https://doi.org/10.1155/2016/7146341

Gross, G., Huber, G., Klosterkötter, J., & Linz, M. (1987). *Bonn Scale for the Assessment of Basic Symptoms: BSABS.* Berlin, Germany: Springer.

Guillory, S., Velthorst, E., Hamilton, H., Roach, B., Bachman, P., Belger, A., … Mathalon, D. (2018a). Atypical P300, but not MMN, amplitude differentiates conversion patterns in psychosis prodrome with versus without comorbid autism spectrum disorder. *Early Intervention in Psychiatry*. https://doi.org/http://dx.doi.org/10.1111/eip.12722

Guillory, S., Velthorst, E., Hamilton, H., Roach, B., Bachman, P., Belger, A., … Mathalon, D. (2018b). Atypical P300 amplitude differentiates conversion patterns in psychosis prodrome when autism spectrum disorder is comorbid. *Biological Psychiatry*.

Jutla, A., Donohue, M. R., Veenstra-Vanderweele, J., & Foss-Feig, J. (2020). Resting-State Functional Connectivity in Youth With Co-Occurring Autism Spectrum Disorder and Psychotic-Like Symptoms. *Biological Psychiatry*. https://doi.org/10.1016/j.biopsych.2020.02.322

Jutla, A., Donohue, M. R., Veenstra-VanderWeele, J., & Foss-Feig, J. (2020). Reported autism diagnosis strongly predicts psychotic-like experiences in the Adolescent Brain Cognitive Development cohort. https://doi.org/https://doi.org/10.1101/2020.02.07.20021170

Karcher, N. R., Barch, D. M., Avenevoli, S., Savill, M., Huber, R. S., Simon, T. J., … Loewy, R. L. (2018). Assessment of the prodromal questionnaire-brief child version for measurement of self-reported psychoticlike experiences in childhood. *JAMA Psychiatry*. https://doi.org/10.1001/jamapsychiatry.2018.1334

Loewy, R. L., Bearden, C. E., Johnson, J. K., Raine, A., & Cannon, T. D. (n.d.). The prodromal questionnaire (PQ): preliminary validation of a self-report screening measure for prodromal and psychotic syndromes. *Schizophrenia Research*, *79(1)*, 117–125.

Lord, C., Risi, S., Lambrecht, L., Cook, E. H., Leventhal, B. L., Dilavore, P. C., … Rutter, M. (2000). The Autism Diagnostic Observation Schedule-Generic: A standard measure of social and communication deficits associated with the spectrum of autism. *Journal of Autism and Developmental Disorders*. https://doi.org/10.1023/A:1005592401947

Lord, C., Rutter, M., & Le Couteur, A. (1994). Autism Diagnostic Interview-Revised: A revised version of a diagnostic interview for caregivers of individuals with possible pervasive developmental disorders. *Journal of Autism and Developmental Disorders*. https://doi.org/10.1007/BF02172145

Maat, A., Therman, S., Swaab, H., & Ziermans, T. (2020). The Attenuated Psychosis Syndrome and Facial Affect Processing in Adolescents With and Without Autism. *Frontiers in Psychiatry*. https://doi.org/10.3389/fpsyt.2020.00759

McGlashan, T., Walsh, B., & Woods, S. (2010). (2019). *The psychosis-risk syndrome: handbook for diagnosis and follow-up.* (O. U. Press., Ed.). Oxford University.

McGlashan TWB, W. S. (2010). *The Psychosis-Risk Syndrome: Handbook for Diagnosis and Follow-up.* (O. University, Ed.). Oxford, United Kingdom.

Miller, T. J., McGlashan, T. H., Rosen, J. L., Somjee, L., Markovich, P. J., Stein, K., & Woods, S. W. (2002). Prospective diagnosis of the initial prodrome for schizophrenia based on the structured interview for prodromal syndromes: Preliminary evidence of interrater reliability and predictive validity. *American Journal of Psychiatry*. https://doi.org/10.1176/appi.ajp.159.5.863

Moher, D., Liberati, A., Tetzlaff, J., & Altman, D. G. (2009). Preferred reporting items for systematic reviews and meta-analyses: The PRISMA statement. *BMJ (Online)*. https://doi.org/10.1136/bmj.b2535

Schultze-Lutter, F., Ruhrmann, S., Fusar-Poli, P., Bechdolf, A., G. Schimmelmann, B., & Klosterkotter, J. (2012). Basic Symptoms and the Prediction of First-Episode Psychosis. *Current Pharmaceutical Design*. https://doi.org/10.2174/138161212799316064

Schultze-Lutter, F., & Theodoridou, A. (2017). The concept of basic symptoms: its scientific and clinical relevance. *World Psychiatry*. https://doi.org/10.1002/wps.20404

Solomon, M., Olsen, E., Niendam, T., Ragland, J. D., Yoon, J., Minzenberg, M., & Carter, C. S. (2011). From lumping to splitting and back again: Atypical social and language development in individuals with clinical-high-risk for psychosis, first episode schizophrenia, and autism spectrum disorders. *Schizophrenia Research*. https://doi.org/10.1016/j.schres.2011.03.005

Solomon, M., Ozonoff, S., Carter, C., & Caplan, R. (2008). Formal thought disorder and the autism spectrum: Relationship with symptoms, executive control, and anxiety. *Journal of Autism and Developmental Disorders*. https://doi.org/10.1007/s10803-007-0526-6

Sprong, M., Becker, H. E., Schothorst, P. F., Swaab, H., Ziermans, T. B., Dingemans, P. M., … van Engeland, H. (2008). Pathways to psychosis: A comparison of the pervasive developmental disorder subtype Multiple Complex Developmental Disorder and the ‘At Risk Mental State’. *Schizophrenia Research*. https://doi.org/10.1016/j.schres.2007.10.031

Stroup, D. F., Berlin, J. A., Morton, S. C., Olkin, I., Williamson, G. D., Rennie, D., … Thacker, S. B. (2000). Meta-analysis of observational studies in epidemiology: A proposal for reporting. *Journal of the American Medical Association*. https://doi.org/10.1001/jama.283.15.2008

Wilson, C. S., Anthony, L., Kenworthy, L., Fleischman, R., Demro, C., Andorko, N., … Schiffman, J. (2020). Feasibility of psychosis risk assessment for adolescents diagnosed with autism. *Autism*. https://doi.org/10.1177/1362361320909173

Yung, A. R., Yuen, H. P., McGorry, P. D., Phillips, L. J., Kelly, D., Dell’Olio, M., … Buckby, J. (2005). Mapping the onset of psychosis: The Comprehensive Assessment of At-Risk Mental States. *Australian and New Zealand Journal of Psychiatry*. https://doi.org/10.1111/j.1440-1614.2005.01714.x
